# Cross-Cultural Adaptation and Validation of the Danish Version of Inventory of Hyperacusis Symptoms

**DOI:** 10.3390/audiolres15040083

**Published:** 2025-07-04

**Authors:** Susanne Steen Nemholt, Camilla Helge, Polly Scutt, David M. Baguley, Jesper Hvass Schmidt

**Affiliations:** 1 Centre for Communication Disorders in the Capital Region of Denmark, 2900 Hellerup, Denmark; 2 Brain Research–Inter-Disciplinary Guided Excellence, Department of Clinical Research (BRIDGE), Faculty of Health Science, University of Southern Denmark, 5230 Odense, Denmark; 3 Audiology and Speech-Language Pathology, Department of Nordic Studies and Language Sciences, University of Copenhagen, 2300 Copenhagen, Denmark; 4 National Institute for Health Research (NIHR), Nottingham Biomedical Research Centre, Nottingham NG7 2RD, UK; 5 Hearing Sciences, Mental Health and Clinical Neurosciences, School of Medicine, University of Nottingham, Nottingham NG7 2RD, UK; 6 Research Unit for ORL–Head & Neck Surgery and Audiology, Odense University Hospital, 5230 Odense, Denmark; 7 Open Patient Data Explorative Network, Institute of Clinical Research, University of Southern Denmark, 5230 Odense, Denmark

**Keywords:** hyperacusis, Inventory of Hyperacusis Symptoms (IHS), test–retest validation, cultural adaptation, Danish

## Abstract

**Background/Objectives**: This study aimed to (i) cross-culturally adapt the Inventory of Hyperacusis (IHS) into Danish and (ii) assess its usability, validity, and reliability in Danish adults with hyperacusis. **Methods**: The translation followed established guidelines for adapting hearing-related questionnaires. A two-phase design ensured linguistic and cultural adaptation and evaluated test–retest reliability and construct validity. The IHS, consisting of 25 items, was translated and tested in seven participants through cognitive debriefing. In phase two, temporal consistency was assessed in 32 patients. **Results**: Thirty-two participants (twenty-eight female; mean age 49.8 years) completed the study over 2–4 weeks (mean 22 days). Eight used hearing aids, and twenty-four reported tinnitus. The Danish IHS showed good reliability (Cronbach’s alpha = 0.95) and acceptable test–retest reliability, except for the General Loudness factor. While no systematic score changes occurred, significant variability in score changes were noted. **Conclusions**: The Danish IHS appears to be a reliable and valid tool for assessing hyperacusis. Further research is needed, but the IHS-DK shows potential as an effective clinical and research tool for evaluating hyperacusis impact and treatment outcomes.

## 1. Introduction

There is no universally accepted definition for reduced sound tolerance, and different definitions emphasize various aspects of the phenomenon. Sometimes, reduced sound tolerance and sound sensitivity are considered synonymous; other times, sound sensitivity specifically refers to hyperacusis [1]. Terms like “decreased sound tolerance,” “sound sensitivity,” and “sound intolerance” are often used as umbrella terms for several conditions, including misophonia, recruitment, and phonophobia, which should not be confused with hyperacusis [2,3,4,5]. In this article, hyperacusis is defined as a reduced tolerance to sound(s) that are perceived as normal to the majority of the population or were perceived as normal to the person before their onset of hyperacusis [5]. Hyperacusis is often present in association with tinnitus, but much of the literature on tinnitus and hyperacusis consists of studies performed in tinnitus clinics with people seeking help for their condition, and might not be representative of the general population [6]. The prevalence of hyperacusis in persons with tinnitus has been reported to be approximately 40%, with about 25% of tinnitus patients being more bothered by their hyperacusis than by their tinnitus [7]. Nolan et al. [8] note conflicting findings on hyperacusis prevalence by gender, with some studies indicating higher rates in females [9], while others suggest a male prevalence [10,11]. Their research seeks to clarify this relationship by controlling for gender and age as confounding variables, ultimately revealing a higher prevalence of hyperacusis in females, which contrasts with earlier studies.

There are no widely agreed-upon guidelines for the management of hyperacusis [12]. Likewise, the evidence base for clinical interventions for hyperacusis is poor, although a preliminary analysis on the application of cognitive behavioural therapy (CBT) for patients suffering from misophonia, hyperacusis, and tinnitus suggests that CBT can lead to meaningful improvements in a patient’s quality of life and their ability to cope with their condition [13].

There are no objective ways to determine the severity of hyperacusis, but self-reported hyperacusis questionnaires have been developed in different countries. Owing to concerns about the overall readability of self-report hyperacusis questionnaires, a comparative study of clinically pertinent self-report hyperacusis questionnaires investigated this and the variability in readability of single items within each questionnaire [14]. Questionnaires were included if they (1) focused on quantifying and characterizing an individual’s sound tolerance difficulties, (2) were designed to be completed by the patient without help or guidance from a clinician, (3) were used or intended for use in the clinic, and (4) had undergone psychometric validation [14].

Five questionnaires were included: The Hyperacusis Questionnaire (HQ) [15], Geräushüberempfindlichkeits-Fragebogen (GÛF) [16], Noise Avoidance Questionnaire (NAQ) [17], Sound Sensitive-Tinnitus Index (SSTI) [18], and Inventory of Hyperacusis Symptoms (IHS) [19].

The HQ was developed in France and is one of the most clinically used questionnaires; it has been translated, adapted, and validated into many languages [20,21,22]. The GÜF was initially validated among patients with tinnitus and hyperacusis (n = 226) in a German population [16]. A further validation was carried out with the English translation of the questionnaire [23]. All patients (n = 91) were diagnosed with tinnitus and hyperacusis. Although they found a good internal consistency (Cronbach’s alpha = 0.92), the factorial structure of the original GÜF was not completely supported by the results in this study. Furthermore, the translation process of the questionnaire from German to English was not reported, which can affect the linguistic, socio-cultural, and pragmatic understanding. The same goes for the noise-avoidance questionnaire, NAQ [17], and to some degree for HQ [14]. SSTI and IHS were developed in English and are both validated [18,19]. While SSTI was developed to measure comorbid tinnitus and hyperacusis [18], the purpose of the IHS is to measure symptom severity, treatment outcomes, and diagnostic differentiation of hyperacusis [19].

Margol-Gromada et al. [14] used four well-established readability formulas, with reading levels calculated by each formula and overall readability as well. They concluded that the readability for all five questionnaires was written at close to or exceeding the recommended reading grade levels. According to the American Medical Association (AMA), the reading grade level of medical questionnaires should correspond to 5th to 6th grade to ensure the accessibility, whereas the U.S. National Institutes of Health (NIH) recommends a 7th to 8th grade reading level [14]. They recommended that developers of the questionnaires improve readability by using mono- or bi-syllabic word, avoiding medical terms, using shorter sentences, and avoiding including too much content within a single item.

The IHS questionnaire had an average sentence length of 10.2 words (mean 11.08) and relatively few complex and long words. Furthermore, the single-item analysis showed that the IHS was the questionnaire with the fewest items with a readability level above the recommended level: 68% of the items scored a readability level below the maximum recommended 8th grade level. The single-item analysis also revealed that a grade level of 16 was recommended for item 11, suggesting reading proficiency at a college level to understand this single item [14]. Therefore, this one item, item 11, must be considered when applying IHS.

The IHS questionnaire was developed by Greenberg and Carlos [19] to serve as a hyperacusis measurement tool in research and clinical practice. The IHS consists of 25 items with response choices on a 4-point Likert scale (response options: not at all, a little, somewhat, and very much so) with a maximum score of 100. Items are grouped into five dimensional factors: Psychosocial Impact (nine items: no. 12–16 and 22–25), Emotional Arousal (five items: no. 3–6 and 21), Functional Impact (six items: no. 7–11 and 18), General Loudness (three items: no. 1–2 and 21), and Communication (two items: no. 19 and 20). A cut-off score of ≥69 indicating the likelihood of clinically significant hyperacusis, followed by severe (≥80) and very severe (≥89), has been suggested by the developer of the IHS. Data from 450 participants (mean age of 35 years, SD = 1.6 years) were used to assess the psychometric properties of the IHS. Participants were members of online tinnitus and hyperacusis support groups from 37 countries. The following questionnaires were completed online: the IHS, the Patient Health Questionnaire-4 (PHQ-4), and the World Health Organization Brief Quality of Life Index (WHOQOLBREF). Self-reported information about hearing loss and tinnitus was optional to provide, and 433 participants reported data on the presence and severity of both.

A further validation of the IHS questionnaire was presented in a retrospective study looking at data from 100 patients (mean age 55 years, SD = 13 years) seeking help for tinnitus and/or hyperacusis from an audiology clinic in the United Kingdom [24]. Besides IHS, the data included the following questionnaires: Tinnitus Handicap Inventory (THI), HQ, Insomnia Severity Index, Generalized Anxiety Disorder, and Patient Health Questoinnaire-9. Audiological measures were the pure-tone average threshold (PTA) and uncomfortable loudness levels (ULL). The latter was used in combination with HQ to identify the participants with hyperacusis. Based on the HQ criterion, 43% of the patients were categorized as having hyperacusis, whereas the ULL values indicated hyperacusis in 28% of the patients (at least one ULL value of ≤77 dB HL or below). Five patients were diagnosed with severe hyperacusis (ULL values of <30 dB HL at any frequency) [24]. With this as a reference, the total IHS score was significantly different between patients diagnosed with and without hyperacusis supporting the convergent validity of the IHS questionnaire. Due to their results, the authors propose a cut-off score of 56 instead of 69 to gain the highest corresponding sensitivity and specificity [24].

In addition to a good internal consistency between the 25 items and five factors of the IHS (α = 0.96), the PTA (pure tone average) neither correlates with the IHS-total nor the factors, which supports the discriminant validity, as hyperacusis is thought to be independent of hearing loss. Furthermore, the authors found moderate to strong correlations with all the above-mentioned questionnaires (*r* ranging from 0.54 to 0.61), except for the Insomnia Severity Index, suggesting moderate to high convergent validity between those questionnaires and the IHS [24]. This implies that IHS scores are likely to reflect hyperacusis but could also to some degree reflect the co-occurrence of tinnitus, anxiety, and depression.

In sum, recent studies of the IHS have shown promising results of both validity and psychometric properties [25]. Additionally, a systematic review of psychometric properties of measures of sound sensitivity by Kula et al. [26] singled out IHS for its generalizability and internal consistency in both clinical and non-clinical populations, whereas the HQ, in comparison, is internally consistent for just the general population. Furthermore, the authors of the systematic review suggest that further studies include more reliability measure, including test–retest reliability, to gain a more comprehensive picture of the questionnaire’s reliability.

With this and the results from the single item analysis in Margol-Gromada et al. [14] concerning item 11 in mind, we decided to translate and cross-culturally adapt the Inventory of Hyperacusis Symptoms (IHS) developed by Greenberg et al. [19].

The overall aim of the present study is to perform a Danish cross-cultural adaption of the IHS. A Danish version of the IHS will allow the assessment of hyperacusis specific to this population. The objective of this study is (i) to translate and cross-culturally adapt the IHS into Danish and (ii) to investigate its usability, validity, and reliability for adults Danes with hyperacusis.

## 2. Materials and Methods

We conducted a two-phase study to translate and culturally adapt the IHS into Danish, as well as to evaluate its clinometric properties (test–retest reliability and convergent construct validity). In the first phase, the IHS questionnaire was translated into Danish and administered to seven participants, followed by cognitive debriefing to investigate the participants’ comprehension of the questions. In the second phase, the adjusted questionnaire was tested for consistency over time in a small sample of patients (n = 32). Written informed consent was obtained from all participants.

### 2.1. Phase 1—Translation and Cross-Cultural Adaptation

**Figure 1:**
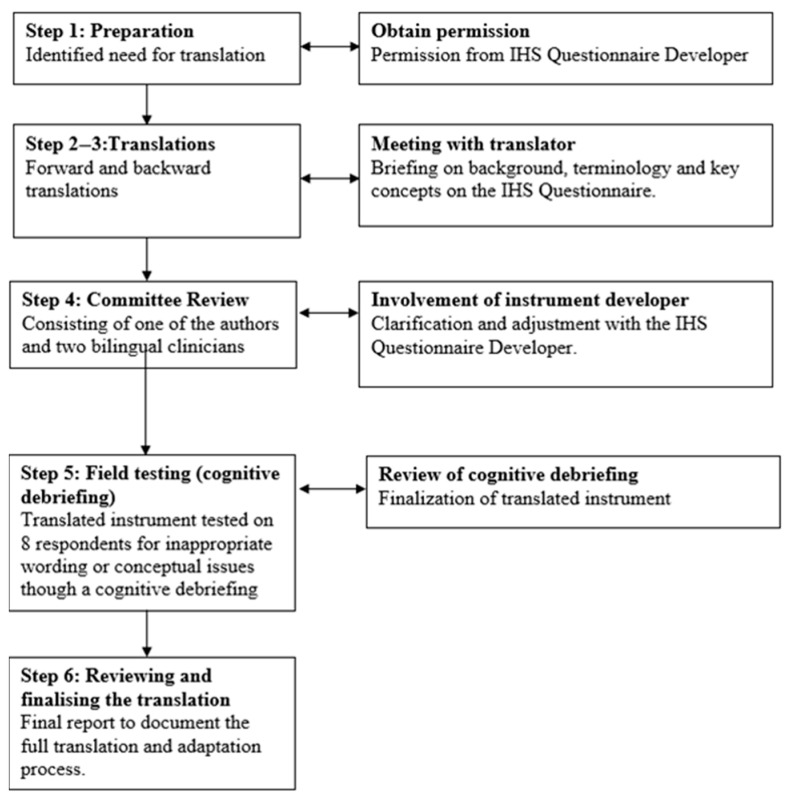
Flowchart of the Danish translation and cross-cultural adaptation process of the Inventory of Hyperacusis Symptoms following the 6 step-by-step Good Practice Guide for Translating and Adapting Hearing-Related Questionnaires for different languages and cultures.

The 6 step-by-step Good Practice Guide for Translating and Adapting Hearing-Related Questionnaires for different languages and cultures [27] was selected as the most relevant best practice guidelines to follow for Phase 1. The six steps from the international guidelines were followed. Figure 1 shows the process.

#### 2.1.1. Step 1: Preparation

The first step (item 1a) in the preparation was to identify whether a Danish version of IHS already existed. No versions were found within the Danish audiological scientific or clinical community. The next step (item 1b) involved gaining permission to use and translate the questionnaire. The original developer of the IHS, Dr. Ben Greenberg, was contacted, and he granted permission for the IHS to be translated; he was also invited to be involved in the process (item 1c). To accommodate the needs of end users (item 1d), the layout of the origin was adjusted.

#### 2.1.2. Step 2–3: Translation

Due to time constraints, the forward and back translation were performed by two professional translators. First, a professional native Danish translator, knowledgeable of the English-speaking culture and living in Denmark, was recruited to translate the IHS-questionnaire from English into Danish (item 2a). The translator was briefed on the background, terminology, and key concepts of the IHS by one of the authors (S.S.N.) (item 2b and 2c). The translator was also instructed to focus on conceptual rather than literal translations and to use natural, simple, and concise language for the broadest audience (world-for-world not word-for-word) (item 2d and 2e). This process resulted in a Danish version of the IHS, which was then back translated into English by a professional native English translator who was living in London but had lived in Denmark for 20 years and possessed knowledge of both the Danish language and culture (step 3a). The translator was independent and had no knowledge of the original instrument (step 3b). After the translation process, the original IHS and the two translations were compared to assess whether any discrepancies were present among them (item 3c).

#### 2.1.3. Step 4: Committee Review

An expert panel (committee review) consisting of one of the authors (S.S.N.) and two trained clinical audiologists, each with over ten years of experience in the field of audiology was established (item 4a).

The purpose of the committee review was to achieve cross-cultural equivalence in the forward translation. Any discrepancies between the original IHS and the two translations were discussed and resolved in the meeting. After reviewing each questionnaire item, the committee members agreed on the most adequate translation. Table 1 provides examples of the process. Any unresolved discrepancies were formulated and sent to the author of the original IHS for clarification and adjustment (item 4b). Item 11 was particularly focused on during the committee review; the reviewers found the meaning of the sentence unclear, and the readability study had identified the item difficult to read. In agreement with the developer of the IHS, the wording of item 11 was change from “My sensitivity to sounds can make it difficult to maintain important work, academic, and/or household responsibilities” to “My sensitivity to sounds can make it difficult to maintain important tasks at work and/or at home”.

This resulted in a new suggestion for a Danish IHS which was back translated to English to confirm equivalence and cross-referenced with the original instrument until a satisfactory version was reached.

#### 2.1.4. Step 5: Field Testing

There is no consensus on the desired sample size for the field testing, but the good practice guidelines recommend using a sample size of eight participants for pre-testing. However, due to the COVID-19 lockdown that coincided with the field-testing period, a cognitive debriefing of the final Danish IHS (field testing) was conducted with seven participants who had hyperacusis, aged 20.9 to 76.1 years (mean age = 49.8, SD = 12.7 years). These participants were recruited from the Centre for Communication Disorders (CCD) (item 5a). During the session, they were asked to complete the instrument while “thinking aloud” and provide explanations for each of their responses. If any word, phrase, or question was misinterpreted or if the participant did not understand it, it was noted how the researcher (S.S.N.) clarified them. Participants were also asked if any items were irrelevant to them or if any questions made them feel uncomfortable. The purpose of the pilot study was to determine whether the instrument items were intelligible and easily understood by the public. The cognitive debriefing was conducted with the target audience to ensure that they understood the questions and that the questions were culturally appropriate. An updated version of the questions, with feedback from the cognitive debriefing process, was sent to the author of the original IHS. Table 2 shows the questions and answers for this correspondence. Any dubious items were further adjusted (by S.S.N.).

#### 2.1.5. Step 6: Reviewing and Finalizing the Translation

A few modifications were made to the words/phrases incorporated, but none of the items were offensive or uncomfortable (item 5b). Also, no questions were removed due to irrelevance (see Appendix A for the translation and adaptation process and Appendix B for the Danish version of the Inventory of Hyperacusis Symptoms (IHS)). The final version was used in the second phase of the study.

### 2.2. Phase 2—Measures and Statistical Analyses

#### 2.2.1. Procedure and Test Population

In the second phase of the study, a total of 32 participants aged 20.9 to 76.1 years (mean age = 49.8, SD = 12.7 years) were recruited from the CDD. Of these, 87.5% (n = 28) were female. Detailed demographic data is provided in the result section, specifically in Table 3. All participants provided voluntary consent for participation and were recruited through an announcement displayed in the lobby of the CCD. Each participant had been referred from an Ear–Nose–Throat (ENT) clinic or Audiological Department indicating hyperacusis, accompanied by an audiogram and medical assessment. To authentically represent the patient population encountered in clinical contexts, no individuals were excluded based on other diagnoses. Prior to enrollment in the study, it was required that sound-therapy and/or other forms of intervention had begun more than three months earlier. All participants completed a written informed consent form prior to their inclusion in the study.

#### 2.2.2. Material

Participants were requested to complete a questionnaire on two separate occasions. Following the completion of the questionnaire at Time 1 (T1), participants were sent either an electronic email or a postal version of the same questionnaire two weeks later at Time 2 (T2). A reminder via email or telephone was conducted for those who had not submitted their responses approximately one week after dispatching the T2 questionnaire. Data collection occurred from 27 May 2020 to 18 January 2021, with a mean interval of 22 days (ranging between 2 and 4 weeks) between T1 and T2. Audiograms and diagnostic data provided by the referring physician were available for all participants. We calculated each participant’s average hearing ability by taking the mean of thresholds at 500, 1000, 2000, and 4000 Hz.

Participants diagnosed with tinnitus were assessed for severity using the Danish version of the Tinnitus Handicap Inventory (THI) [28,29]. THI scores range from 0 to 100 and categorize severity into five levels: very mild (0–18), mild (18–36), moderate (38–56), severe (58–76), and catastrophic (78–90). The THI was designed to evaluate treatment efficacy in clinical practice and consists of 25 items, which can be further divided into three subscales that reflect the emotional state (emotional), the ability to function in daily life (functional), and the extent of catastrophic thinking (catastrophic) experienced by the patient. Notably, the THI-DK was the sole reliable and valid Danish tinnitus questionnaire available at the time this study was conducted; however, subsequent to this study, Hald et al. [30] introduced a translation and validation of the Tinnitus Functional Index.

To evaluate the severity of hyperacusis among participants, we applied two different classification systems based on the total IHS-dk scores. The first classification system, proposed by Greenberg and Carlos [19], categorizes hyperacusis using cut-offs of 69, 80, and 89 for the presence of, severe, and very severe cases, respectively. The second, a modified system validated by Aazh, Danesh, and Moore [24], adjusts the lower threshold to 56 for identifying mild to moderate hyperacusis while retaining the same thresholds for severe and very severe cases. This dual approach allowed for a comprehensive assessment of hyperacusis severity across different classification systems, thereby enhancing the robustness of our findings.

#### 2.2.3. Data Analysis

Statistical analyses were conducted using STATA version BE 17.0 for Mac. To ascertain the reliability, we calculated internal consistency reliability coefficients (Cronbach’s alpha) for the IHS-total scale as well as for the five factors inherent within the IHS. In the event of missing data, we reported the quantity of missing cases and conducted a complete case analysis to ensure that our findings were not unduly influenced by incomplete data.

To evaluate the overall test–retest reliability of the measures, the Pearson Correlation Coefficient was employed. These statistical methods allowed us to examine the relationship between scores obtained at T1 and T2, thus providing insight into the stability of participants response over time. For testing the reproducibility of scores, Intraclass Correlation Coefficients (ICC) and Cohen’s kappa coefficients for item reproducibility were computed for the test and retest phases. ICCs were derived using a two-way mixed model with absolute agreement, with ICCs greater than 0.70 and Cohen’s kappa values exceeding 0.40 deemed acceptable [31,32].

Diverse interpretations exist regarding the values of the correlation coefficient, r, as well as the consistency measure, α. It is generally accepted that alpha values ranging from 0.70 to 0.95 indicate acceptable internal consistency [33]. Moreover, values of the correlation coefficient, r, are often categorized with the following conventions: 0.1 indicating a small effect, 0.3 representing a medium effect, and 0.5 denoting a large effect [34].

## 3. Results

### 3.1. Descriptive

A sample of 32 participants (28 females and 4 males, aged 20.9 to 76.1 years and a mean age of 49.8 years) was recruited at the CCD. Comprehensive demographics data are presented in Table 3. All participants reported hyperacusis as their primary concern and were referred on this diagnosis from their ENT. Among the participants, 8 utilized hearing aids, while 24 experienced tinnitus as assessed though their THI scores, categorized as follows: moderate (n = 5), mild (n = 13), very mild or no handicap (n = 6).

The distribution of hyperacusis severity among participants, as assessed by the IHS-dk score, is detailed in Table 4, which compares the findings from two classification systems: Greenberg and Carlos and Aazh, Danesh, and Moore. The mean IHS-dk score for the sample was 66.13 with a range of 32 to 93, indicating a spectrum of hyperacusis severity. In the lower ranges, Aazh, Danesh, and Moore identified that 50% of participants (16 individuals) exhibited no hyperacusis, while an additional 25% (8 participants) were classified as having mild to moderate hyperacusis within the 56-68 range.

### 3.2. Reliability

The reliability of the Danish version of the IHS (IHS-DK) was assessed using Cronbach’s alpha, yielding a coefficient of 0.95, which indicates strong internal consistency and reliability. Table 5 presents the reliability coefficients associated with the IHS-DK. Notably, Cronbach’s alpha value for the factor labelled “General Loudness” was observed to be comparatively lower.

### 3.3. Reproducibility

The Pearson Correlation Coefficient comparing the initial IHS test score to the retest score was calculated to be r = 0.93 (*p* < 0.001). A paired *t*-test revealed no statistically significant difference between the test and retest total score (*p* = 0.10). The test–retest reliability was deemed acceptable for most items with Cohen’s kappa values ranging from 0.40 to 0.82. Detailed test–retest reliability coefficient for each IHS item is depicted in Table 6, column 2. Eight items exhibited kappa values below the established threshold; in particular, item 2 had a kappa value of 0.19, while seven additional items had kappa values ranging from 0.25 to 0.40 (item 3, 4, 5, 9, 10, 11, 22). The ICC for reproducibility of the various factor scores is provided in Table 6, column 3, indicating acceptability for four of the factors (with values ranging from 0.75 to 0.92), but deemed inadequate for the factor “General loudness” (0.56).

**Figure 2:**
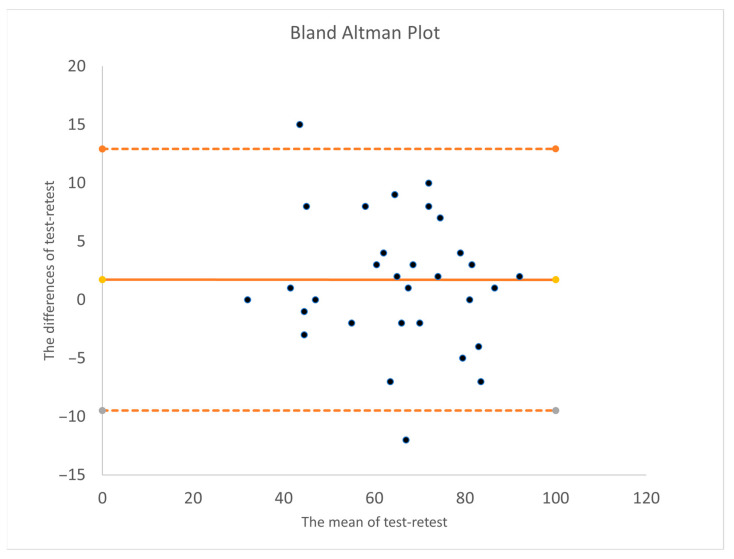
Bland–Altman Plot. The solid line shows the mean difference; dashed lines indicate the limits of agreement (mean ±1.96 standard deviations).

The differences between test and retest scores, along with the mean scores derived from both sessions, are illustrated in Figure 2 utilizing a Bland–Altman Plot. Notably, the analysis reveals no evident systematic changes, with the number of participants exhibiting increased scores equaling those decreased scores between the test and retest administrations. Nevertheless, significant variability is observed, including notable score shifts, suggesting that should this scale to be used in research, any intervention-induced changes measured by this scale would necessitate clear distinction above the observed variability.

## 4. Discussion

The primary objectives of this study were to (i) translate and cross-culturally adapt the IHS questionnaire from English to Danish and (ii) investigate its usability, validity, and reliability for individuals in Denmark experiencing hyperacusis. Initially, the questionnaire underwent cultural linguistic adaptation, which was subsequently tested on seven participants through a cognitive debriefing process. In the following step, the Danish version (IHS-DK) was administered to 32 participants on two separate occasions.

The findings of this investigation indicate that the Danish translation of the IHS (IHS-DK) demonstrates good internal consistency, aligning with previous studies that established similar metrics for the original version and the further validation. This consistency reinforces the notion that the core constructs of hyperacusis can be reliably measured across different cultures and languages.

The internal reliability coefficients for the subscales in our findings are generally lower than those reported by Azha and Moore [24]. They did not explicitly mention the scores of ICC but reported the internal consistency for the IHS and its factors using Cronbach’s alphas with the values: Factor 1 (General Loudness): 0.81, Factor 2 (Emotional Arousal): 0.89, Factor 3 (Psychosocial): 0.92 and Factor 4 (Functional Impact): 0.89. Factor 5 (Communication): Scale reliability coefficient of 0.89. We reported the reliability of the HIS subscales, providing ICC values and kappa statistics for each factor. The ICC values indicate the degree of agreement or consistency in responses across different measurements. Psychosocial, Functional Impact, Communication, and Emotional Arousal were determined to be adequate at 0.92, 0.85, 0.80, and 0.75. The relatively lower Cronbach’s alpha for the General Loudness subscale may reflect a lack of interrelatedness among the items. Given that Cronbach’s alpha is highly sensitive to the number of items included in a subscale, this result is likely attributable to the smaller number of items associated with this dimension. An additional consideration pertains to item 2’s low reproducibility (Cohen’s kappa coefficient = 0.19) and its subscale scores (ICC = 0.56). It appears that the participants provided inconsistent responses to this item between the test and retest phases. This inconsistency may indicate that item 2 is ambiguous or, as suggested by the results presented in Figure 2, could be partially explained by the dynamic or fluctuating nature of hyperacusis. The limitations associated with sound intensity tolerance can fluctuate based on contextual factors, emotional state, stress levels, and mood, which inevitably change over the two to four-week interval between testing sessions.

An important factor to consider is that hyperacusis frequently co-occurs with various comorbid conditions such as affective disorders, tinnitus, post-concussive syndrome (PCS), and hearing loss. These comorbidities may significantly influence an individual’s subjective experience of hyperacusis, potentially varying on a weekly or daily basis, which, in turn, could affect the IHS score and, by extension, the test–retest reliability. This limitation is inherent to this study, akin to other test–retest studies, and underscores the need for caution when employing the IHS to assess the impact of hyperacusis intervention. It is crucial to acknowledge that certain aspects of the IHS-DK scores may be influenced by external factors such as emotional state and quality of life perceptions. Clinicians should carefully interpret these scores, particularly in contexts involving patients with mixed symptoms, and consider integrating assessments of psychological well-being into their practice to provide a more holistic understanding of each patient’s condition.

Specifically, the General Loudness subscale addresses sound tolerance, with item 1 and 21 framed in more general terms using softer language such as “compared to most people my experience is…” and “I think…”, whereas item 2 refers to the experienced pain and/or physical discomfort associated with reduced sound tolerance, potentially making it more susceptible to recall bias due to the more severe nature of its wording.

According to the recommended cut-off scale of the questionnaire, 16 out of 32 participants were determined not to exhibit clinically significant hyperacusis. This discrepancy may arise from varying definitions of hyperacusis used to ascertain its presence or different approaches to defining its severity among the ENTs or Audiological Departments that referred these individuals. A different cutoff score of 56 has been proposed for diagnosing hyperacusis, as this value was demonstrated to yield the highest overall accuracy in a study conducted by Aazh, Danesh and Moore [24]. The sensitivity and specificity rates of 74% and 82% for the cutoff score of 56 provide a more balanced diagnostic criterion than the sensitivity and specificity of 46% and 91%, respectively, associated with the commonly utilized cutoff score of 69 as suggested by Greenberg and Carlos [19,24]. With the application of this novel cutoff scale, only 8 of 32 participants would fall outside the categorization of clinically significant hyperacusis, thereby enhancing the classification accuracy from 50% to 75%.

Another important limitation is the possibility of selection bias. Different criteria might have been used for the hyperacusis diagnosis at the different clinics referring potential participants. Additionally, the prospect that those who chose to participate may differ in personality from those who did not sign up raises the possibility that our sample may not be representative of the entire group of people experiencing hyperacusis.

## 5. Conclusions

We have demonstrated that the Danish translation of the IHS seems to be a reliable and valid general measure of hyperacusis-related issues. With respect to the subscales, further studies using the IHS-DK and other similar measures are needed to clarify whether specific hyperacusis distress measures can be identified. Our data, however, suggest that the total score of the IHS can be used to quantify the impact of hyperacusis on daily living and serve as a valid outcome measure in the clinical setting and in treatment or rehabilitation studies.

We recommend further evaluation and use of the Danish IHS in both research and clinical settings. We encourage researchers to replicate and extend our validation of the Danish IHS using a larger sample recruited from a hospital or clinical setting, focusing on the factor structure of IHS combined with measures of psychological distress and tinnitus.

## Figures and Tables

**Table 1:** Examples of the Inventory of Hyperacusis Symptoms translation presented to review committee.

Steps	Original IHS	IHS FT	IHS BT	Committee Review Notes	IHS-DK Version 1 for Field Testing
Item 7	My sensitivity to sounds can make it difficult to cope.	Min lydfølsomhed kan betyde, at det er svært for mig at overskue en situation.	My auditive sensitivity can make it difficult for me to retain the overview of a situation.	The English word “cope” was translated into the Danish phrase “at overskue en situation” and was back translated to “retrain the overview of a situation”, which was back translated to “retrain the overview of a situation”, which did not correspond to the definition of “to cope”. The Danish phrase “at håndtere hverdagen” is semantically closer to “cope” and was chosen.	Min lydfølsomhed kan betyde, at det er svært for mig at håndtere hverdagen.
Item 14	My increased sensitivity to sounds can make me feel hopeless.	Min lydfølsomhed kan betyde, at jeg føler mig håbløs.	My auditive sensitivity can mean that I feel desperate.	The Danish word “håbløs” has many semantic meanings, including incompetent. The committee believed the intention of the item was to assess whether a person feels like giving up, which in Danish is translated as “opgivende”.	Min lydfølsomhed kan betyde, at jeg føler mig opgivende.

**Table 2:** Questions, clarifications, and adjustments made with the author of the original IHS.

Steps	Questions Send to BG	Response from BG	Response to BG
Item 11: My sensitivity to sounds can make it difficult to maintain important work, academic, and/or household responsibilities	This question was difficult for the committee to understand. Therefore, they suggested the wording was changed to: My sensitivity to sounds can make it difficult to maintain important tasks at work, and/or at home. Would this still cover the items, and would you be ok with this change?	For question 11, I would suggest adding ‘school’ between work and home, so that it could apply to individuals who are studying, as well.	Thank you, I will add “school” in the sentence.
Item 13: My sensitivity to sounds can make it difficult to take part in meaningful activities I used to enjoy.	The committee understood this as activities a person no longer can enjoy due to the hyperacusis. They were a bit puzzled by the word “meaningful”, and suggested to delete it, so the new wording would be: My sensitivity to sound can make it difficult to take part in activities I used to enjoy, if it is ok with you?	For question 13, I don’t see much harm if the word ‘meaningful’ can be removed, however it is meant to signify having to remove oneself from the situations that give life particular meaning, i.e., spiritual/religious activities, parties, concerts, gatherings, one’s profession, etc. Based on the pilot study and clinical observation, the word is intended to reflect the particular challenge of giving up the few activities that define one’s sense of identity and connection in the world.	Thank you for clarifying this. In the Danish pilot, the participants didn’t understand this intention with the question. Maybe it is due to culture differences between our countries. I will try to find a way to include this meaning in the Danish wording without changing the wording too much.
Before item 14–18: My increased sensitivity to sounds can make me feel:	The committee suggested deleting the word “increased” to keep the same terminology though the questionnaire, so the new wording would be: My sensitivity to sounds can make me feel: if it is ok with you?	Taking out the word ‘increased’ for items 14–18 makes sense to me.	
Before item 19–25: I find the challenges of being exposed to loud sounds:	The participants in the pilot found these questions difficult to understand.	For items 19–25, perhaps it could add some clarity to change the preface to “I find the challenges of my sensitivity to sounds:”? Or something similar?	Thank you. I think this will make it easier to read.

**Table 3:** Demographics data.

	Numbers (n)	Percentage (%)	Mean	Range	Median
Patients	32				
Gender (female)	28	87.5			
Age			49.8	20.93–76.11	
Hyperacusis	32	100			
IHS score			66.13	32–93	68.5
Tinnitus	24	75			
THI score			26.2	4–46	26
PTA	31		19.6	0–66.9	
Hearing aid	8				
Other diagnoses					
PCS	11	34.4			
Apoplexy	2	6.25			
ADHD	1	3.125			
ASF	1	3.125			

PCS, Post-concussion syndrome; ADHD, Attention Deficit Hyperactivity Disorder; ASF, Autism Spectrum Disorder.

**Table 4:** Distribution of hyperacusis severity based on IHS-DK scores and classification systems.

Score Range	Greenberg & Carlos (2018) [19]	% (n)	Aazh, Danesh & Moore (2021) [24]	% (n)
<56	Not Applicable	50 (16)	No hyperacusis	25 (8)
56–68	Not Applicable		Mild–moderate hyperacusis	25 (8)
69–79	Hyperacusis	28.1 (9)	Mild–moderate hyperacusis	28.1 (9)
80–88	Severe hyperacusis	18.8 (6)	Severe hyperacusis	18.8 (6)
≥89	Very severe hyperacusis	3.1 (1)	Very severe hyperacusis	3.1 (1)

**Table 5:** Reliability coefficients (Cronbach’s alpha) for the Danish translation of the IHS (IHS-DK, IHS-DK (retest)), and the original version (IHS-US).

Factor	IHS-DK	IHS-DK (Retest)	IHS-US
IHS-total (25 items)	0.95	0.96	0.93
General Loudness	0.66	0.79	
Emotional arousal	0.84	0.88	
Psychosocial	0.90	0.87	
Functional impact	0.91	0.88	
Communication	0.77	0.78	

**Table 6:** Intraclass Correlation Coefficients by subscale.

	Item	Kappa	ICC [95% CI]	Kappa	ICC [95% CI]
	Factor General Loudness		0.67 [0.43,0.83]		0.56 [0.26,0.76]
1	Compared with most people, my experience is that everyday sounds can seem too loud for me.	0.42		0.41	
2	Sounds can cause me to feel pain and/or physical discomfort	0.24		0.19	
21	I think that the challenges of being exposed to loud noises can make it hard for me to be in where there are loud noises	0.54		0.49	
	Factor Emotional Arousal		0.80 [0.63,0.90]		0.75 [0.55,0.87]
3	When I hear loud sounds, it can make me stressed	0.41		0.36	
4	When I hear loud sounds, it can make me tense	0.34		0.29	
5	When I hear loud sounds, it can make me angry	0.34		0.28	
6	When I hear loud sounds, it can make me irritated	0.62		0.64	
17	My auditive sensitivity can mean that I feel frustrated	0.41		0.42	
	Factor Psychosocial		0.93 [0.86,0.96]		0.92 [0.83,0.96]
12	My auditive sensitivity can mean it is difficult for me to lead the social life I would like to	0.68		0.64	
13	My auditive sensitivity can mean it is difficult for me to take part in activities that have previously been important to me	0.57		0.58	
14	My auditive sensitivity can mean that I feel desperate	0.51		0.49	
15	My auditive sensitivity can mean that I feel alone or isolated	0.59		0.56	
16	My auditive sensitivity can mean that I feel afraid	0.69		0.68	
22	I think that the challenges of being exposed to loud noises can make it harder to use transport (car, buses, trains, bicycle, motorcycle etc.)	0.39		0.35	
23	I think that the challenges of being exposed to loud noises can make me afraid to leave my home for fear of being exposed to loud noises	0.82		0.82	
24	I think that the challenges of being exposed to loud noises can have made getting around a greater problem	0.60		0.57	
25	I think that the challenges of being exposed to loud noises can make it hard for me to do the things I used to be able to enjoy	0.53		0.54	
	Factor Functional Impact		0.87 [0.75,0.94]		0.85 [0.71,0.93]
7	My auditive sensitivity can mean it is difficult for me to cope with everyday life	0.55		0.50	
8	My auditive sensitivity can mean it is difficult for me to concentrate	0.68		0.65	
9	My auditive sensitivity can mean it is difficult for me to relax	0.27		0.25	
10	My auditive sensitivity can mean it is difficult for me to sleep	0.44		0.40	
11	My auditive sensitivity can mean it is difficult for me to manage important tasks at work, school and/or in the house	0.39		0.34	
18	My auditive sensitivity can mean that I feel tired or exhausted	0.53		0.53	
	Factor Communication		0.83 [0.69,0.92]		0.80 [0.62,0.90]
19	I think that the challenges of being exposed to loud noises can be hard to explain to friends and family	0.49		0.44	
20	I think that the challenges of being exposed to loud noises can be difficult to explain to doctors and other specialists	0.48		0.45	

## Data Availability

The data presented in this study are available on request from the corresponding author due to privacy concern.

## Abbreviations

The following abbreviations are used in this manuscript:

IHS	Inventory of Hyperacusis
CBT	Cognitive Behavioural Therapy
HQ	The Hyperacusis Questionnaire
GÜF	Geräushüberempfindlichkeits-Fragebogen
NAQ	Noise Avoidance Questionnaire
SSTI	Sound Sensitive-Tinnitus Index
AMA	American Medical Association
NIH	U.S. National Institutes of Health
PHQ-4	Patient Health Questionnaire-4
WHOQOLBREF	World Health Organization Brief Quality of Life Index
THI	Tinnitus Handicap Inventory
PTA	Pure-Tone Average threshold
ULL	Uncomfortable Loudness Levels
CCD	Centre for Communication Disorders
ENT	Ear Noise Throat
ICC	Intraclass Correlation Coefficients
PCS	Post-Concussion Syndrome
ADHD	Attention Deficit Hyperactivity Disorder
ASF	Autism Spectrum Disorder

## Appendix A

**Table A1:** Translation and adaptation process.

Iten no:	Source Language (Country and Version)	Forward TR New Language	Back Translation	Resolution Reasoning	Updated Forward Translation (January 2020)	Updated Back Translation (January 2020)
Title of instrument	Inventory of Hyperacusis Symtoms (IHS)	Registrering af symptomer på lydfølsomhed (IHS)	Recording the symptoms of auditive sensitivity (IHS)	N/A	N/A	N/A
Response options	(1) not at all, (2) a little, (3) somewhat (4) very much so	(1) slet ikke, (2) i nogen grad, (3) ret meget, (4) virkelig meget	(1) not at all, (2) to some extent, (3) quite a lot, (4) a great deal	The Danish translation distorts the scale towards a more severe hyperacusis experience.	(1) slet ikke, (2) i mindre grad (3) i nogen grad, (4) i høj grad	(1) not at all, (2) to a small extent, (3) to some extent, (4) to a great extent
Instruction	Please answer each item to the best of your ability, as close to your experience as possible:	Besvar nedenstående udsagn efter bedste evne og så tæt på din egen erfaring som muligt:	Answer the statements below to the best of your ability and as closely as possible to your own experience.	The Danish words “erfaring” and “oplevelse” both mean experience. However, the word “oplevelse” is used more as an everyday word, whereas “erfaring” also could mean professional experience. Furthermore, the word “oplevelse” occurs thought the questionnaire and is better for continuity.	Besvar nedenstående udsagn efter bedste evne og så tæt på din egen oplevelse som muligt.	Answer the statements below to the best of your ability and as closely as possible to your own experience.
1	Compared to most people, common everyday sounds seem excessively loud to me.	Sammenlignet med de fleste personer oplever jeg, at almindeligt forekommende lyde kan være for kraftige for mig.	Compared with most people, my experience is that commonly occurring sounds can be too loud for me.	The Danish word for everyday sounds would be “hverdagslyde”, which was lost in the translation. The word “virke” corresponds better to the word “seem” than the word “kan være”, which mens “can be”: the sounds are not louder, but can be experienced as (seem) louder.	Sammenlignet med de fleste personer oplever jeg, at hverdagslyde kan virke for kraftige for mig.	Compared with most people, my experience is that everyday sounds can seem too loud for me.
2	Sound can cause me pain and/or physical discomfort	Lyde kan få mig til at føle smerte og/eller fysisk ubehag.	Sounds can cause me to feel pain and/or physical discomfort.	N/A	N/A	Sounds can cause me to feel pain and/or physical discomfort. (N/A)
	**Hearing loud sounds can make me feel:**	**Når jeg hører kraftige lyde, kan det gøre mig:**	**When I hear loud sounds, it can make me:**	**N/A**	**N/A**	**When I hear loud sounds, it can make me (N/A)**
3	stressed out	stresset	stressed	N/A	N/A	stressed (N/A)
4	tense	anspændt.	tense	N/A	N/A	tense (N/A)
5	angry	vred.	angry	N/A	N/A	angry (N/A)
6	irritated	irritabel.	irritable	The word was not translated correctly. Irritated not irritable	irriteret.	irritated
	**My sensitivity to sounds can make it difficult:**	**Min lydfølsomhed kan betyde, at det er svært for mig:**	**My auditive sensitivity can make it difficult for me:**	**N/A**	**N/A**	**My auditive sensitivity can mean it is difficult for me:**
7	to cope	at overskue en situation.	to retain the overview of a situation.	The English word to “cope” was back translated to “retrain the overview of a situation”, which does not correspond well with the definition of “to cope”. The phrase “håndtere hverdagen” is more likely semantically closer to “cope”.	at håndtere hverdagen.	to cope with everyday life
8	to concentrate	at koncentrere mig.	to concentrate.	N/A	N/A	to concentrate (N/A)
9	to relax	at slappe af.	to relax.	N/A	N/A	to relax (N/A)
10	to sleep	at sove.	to sleep.	N/A	N/A	to sleep (N/A)
11	to maintain important work, academic, and/or household responsibilities	at udføre større arbejdsopgaver, at tænke abstrakt og/eller udføre huslige pligter.	to perform substantial tasks, think in abstract terms and/or perform domestic duties.	“To maintain important work” is less readable than “to work”, and “academic responsibilities” less readable than “to study”.	at klare vigtige opgaver på arbejdet, og/eller i hjemmet	to manage important tasks at work and/or in the home.
12	to have the social life I wish to have	at leve det sociale liv, jeg ønsker	to lead the social life I want to.	The phrase becomes more informal.	at leve det sociale liv, jeg gerne vil	to lead the social life I would like to
13	to take part in meaningful activities I used to enjoy	at deltage i relevante aktiviteter, som ellers har haft betydning for mig.	to take part in relevant activities that have otherwise been important to me.	The Danish word “relevante” is redundant to the meaning of the item. The word “tidligere” is clearer than the word “ellers”.	at deltage i aktiviteter, som tidligere har haft betydning for mig.	to take part in activities that have previously been important to me
	**My increased sensitivity to sounds can make me feel:**	**Min lydfølsomhed kan betyde, at jeg føler mig:**	**My auditive sensitivity can mean that I feel:**	**N/A**	**N/A**	**My auditive sensitivity can mean that I feel (N/A)**
14	hopeless	håbløs.	desperate.	The Danish word “håbløs” has many semantic meanings, including being incompetent. We believe the item wants to assess whether a person feels like giving up, which in Danish is translated as “opgivende”.	opgivende.	desperate
15	alone or isolated	alene eller isoleret.	alone or isolated.	N/A	N/A	alone or isolated (N/A)
16	afraid	bange.	afraid.	N/A	N/A	afraid (N/A)
17	frustated	frustreret.	frustrated.	N/A	N/A	frustrated (N/A)
18	tired or fatigued	træt eller udmattet.	tired or exhausted.	N/A	N/A	tired or exhausted (N/A)
	**I find the challenges of being exposed to loud sounds:**	**Når jeg bliver udsat for kraftige lyde, synes jeg, at det kan:**	**When exposed to loud noises, I find it can:**	**The English back translation was too far from the original; it changed the meaning of the item, and lacked the word “challenges”.**	**Jeg synes, at udfordringerne ved at blive udsat for kraftige lyde kan:**	**I think that the challenges of being exposed to loud noises can:**
19	difficult to explain to my friends and family	være svært at forklare situationen over for venner og familie.	be hard to explain the situation to family and friends.	It is not the situation that is hard to explain, but the challenges	være svære at forklare over for venner og familie.	be hard to explain to family and friends
20	difficult to explain to doctors and other care providers	være svært at forklare situatioen over for læger og andre sundhedspersoner.	be hard to explain the situation to doctors and other healthcare professionals.	It is not the situation that is hard to explain, but the challenges. The word “fagpersoner” also covers professionals who are not related to the healthcare system	være svære at forklare over for læger og andre fagpersoner.	be hard to exolain to doctors and other specialists
21	can make it difficult to be in loud places	være svært at opholde mig på steder, hvor der er kraftige lyde.	be hard to stay in places where there are loud noises.	The phrase becomes more informal.	gøre det svært for mig at være steder, hvor der er kraftige lyde	make it hard for me to be in places where thare are loud noises
22	can make it harder to use transportation (car, buses, trains, bicycle, motorbike, etc.)	kan være sværere at bruge transport (bil, busser, tog, cykel, motorcykel osv.)	make it harder to use transport (car, buses, trains, bicycle, motorcycle etc.)	This translation is grammatically better	gøre det sværere at bruge transport (bil, busser, tog, cykel, motorcykel osv.)	make it harder to use transport (car, buses, trains, bicycle, motorcycle etc.)
23	can make me afraid to leave my house for fear I may be exposed to loud sounds	gøre mig bange for at forlade huset, fordi jeg vil undgå at blive udsat for kraftige lyde.	make me afraid to leave the house, because I want to avoid exposure to loud noises.	The English word “fear” in Danish is “frygt”, whereas “vil undgå” means “want to avoid”. Many Danes do not live in a house. It is better translated to “mit hjem” which means “my home” This translation is also grammatically better	gøre mig bange for at forlade mit hjem af frygt for at blive udsat for kraftige lyde.	make me afraid to leave my home for fear of being exposed to loud noises
24	has made it more of a problem to get around	have gjort det til et større problem at komme omkring.	have made getting around more of a problem.	N/A	N/A	have made getting around a greater problem (N/A)
25	can make it difficult to do the things I used to enjoy	gøre det svært for mig at gøre de ting, jeg plejede at gøre.	make it hard for me to do the things I used to do.	The word “to enjoy” is “nyde”, where “to do” is “gøre.	gøre det svært for mig at gøre de ting, jeg plejede at kunne nyde.	make it hard for me to do the things I used to be able to enjoy
Score	Total score (each item rated 1–4): 69 indicates likelihood of hypercausis, 80 severe, 89 very severe.	Samlet score (hvert udsagn bedømt på en skala fra 1 4il 4): 69 angiver sandsynlig forekomst af lydfølsomhed, 80 svær, meget svær.	Total score (each statement assessed on a scale from 1-4): 69 indicates probable occurrence of auditive sensitivity, 80 severe, very severe.	N/A	N/A	Total score (each statement assessed on a scale from 1–4): 69 indicates probable occurrence of auditive sensitivity, 80 severe, very severe (N/A)

N/A =Not applicable.

## Appendix B. [27]

**Table A2:** Danish version of IHS.

Fornavn:				
Efternavn:				
Fødselsdag:				
Dato for udfyldelse:				
**Nedenstående udsagn beskriver nogle af de problemer, man kan opleve i forbindelse med lydfølsomhed.** **Sæt kryds ud for det svar, som passer bedst til din oplevelse. Det er vigtigt, at du besvarer alle spørgsmålene og du må kun sætte ét kryds ud for hvert udsagn**.	**slet ikke**	**lidt**	**i nogen grad**	**ret meget**
1. Sammenlignet med de fleste personer, oplever jeg, at hverdagslyde kan virke for høje for mig.				
2. Lyde kan få mig til at føle smerte og/eller fysisk ubehag.				
3. Når jeg hører høje lyde, kan det gøre mig stresset.				
4. Når jeg hører høje lyde, kan det gøre mig anspændt.				
5. Når jeg hører høje lyde, kan det gøre mig vred.				
6. Når jeg hører høje lyde, kan det gøre mig irriteret.				
7. Min lydfølsomhed kan betyde, at det er svært for mig at håndtere hverdagen.				
8. Min lydfølsomhed kan betyde, at det er svært for mig at koncentrere mig.				
9. Min lydfølsomhed kan betyde, at det er svært for mig at slappe af.				
10. Min lydfølsomhed kan betyde, at det er svært for mig at sove.				
11. Min lydfølsomhed kan betyde, at det er svært for mig at fastholde vigtige opgaver på studiet, arbejdet og/eller i hjemmet.				
12. Min lydfølsomhed kan betyde, at det er svært for mig at leve det sociale liv, jeg gerne vil.				
13. Min lydfølsomhed kan betyde, at det er svært for mig at deltage i aktiviteter, som tidligere har haft betydning for mig.				
14. Min lydfølsomhed kan betyde, at jeg føler mig modløs.				
15. Min lydfølsomhed kan betyde, at jeg føler mig alene eller isoleret.				
16. Min lydfølsomhed kan betyde, at jeg føler mig bange.				
17. Min lydfølsomhed kan betyde, at jeg føler mig frustreret.				
18. Min lydfølsomhed kan betyde, at jeg føler mig træt eller udmattet.				
19. Jeg synes, at udfordringerne ved at blive udsat for høje lyde kan være svære at forklare over for venner og familie.				
20. Jeg synes, at udfordringerne ved at blive udsat for høje lyde kan være svære at forklare over for læger og andre fagpersoner.				
21. Jeg synes, at udfordringerne ved at blive udsat for høje lyde kan gøre det svært for mig at være i støjende omgivelser.				
22. Jeg synes, at udfordringerne ved at blive udsat for høje lyde kan gøre det sværere at bruge transport (bil, busser, tog, cykel, motorcykel osv.)				
23. Jeg synes, at udfordringerne ved at blive udsat for høje lyde kan gøre mig bange for at forlade mit hjem af frygt for at blive udsat for kraftige lyde.				
24. Jeg synes, at udfordringerne ved at blive udsat for høje lyde kan have gjort det til et større problem at komme ud.				
25. Jeg synes, at udfordringerne ved at blive udsat for høje lyde kan gøre det svært for mig at gøre de ting, jeg plejede at kunne nyde.				

Til hørekonsulenten:

Beregn den totale IHS-dk score:

(antal “slet ikke” × 1) + (antal “lidt” × 2) + (antal “i nogen grad” × 3) + (antal “ret meget” × 4)

= ________________

Graduering af den oplevede gene af lydfølsomhed baseret på den totale IHS-dk score ud fra to forskelligt foreslået cut-off:

1. Greenberg and Carlos (2018) (Ref. [19])
  - <69 ingen hyperacusis
  - mellem 69 and 79 hyperacusis
  - mellem 80–88 alvorlig hyperacusis
  - ≥89 meget alvorlig hyperacusis
2. Aazh, Danesh and Moore (2021) (Ref. [24])
  - <56 ingen hyperacusis
  - mellem 56 and 79 mild-moderat hyperacusis
  - mellem 80–88 alvorlig hyperacusis
  - ≥89 meget alvorlig hyperacusis
